# Double Heterozygous CDAN1 Variants of Uncertain Significance Associated With a Phenotype Consistent With Congenital Dyserythropoietic Anemia Type 1

**DOI:** 10.1155/crh/8073716

**Published:** 2026-08-31

**Authors:** Kevin G. Zablonski, Neil A. Lachant, Archibald Perkins, William J. Archibald

**Affiliations:** ^1^ Division of Hematology and Oncology, Wilmot Cancer Center, University of Rochester Medical Center, Rochester, New York, USA, rochester.edu; ^2^ Department of Pathology and Laboratory Medicine, University of Rochester Medical Center, Rochester, New York, USA, rochester.edu

**Keywords:** case report, CDA, CDA Type 1, CDAN1, congenital dyserythropoietic anemia

## Abstract

Congenital dyserythropoietic anemia is a group of hereditary disorders characterized by erythroid hyperplasia and ineffective erythropoiesis, resulting in anemia of varying severity. Congenital dyserythropoietic anemia Type 1 (CDA‐1) is classically associated with biallelic mutations in the CDAN1 gene. Here, we report the first case of compound heterozygous CDAN1 mutations p.(D1043V) and p.(S1036F) in clinical practice, presenting in a 24‐year‐old woman with mild, asymptomatic macrocytic anemia and hyperferritinemia. These variants are currently classified as variants of uncertain significance; however, this report represents the first clinical case of this compound heterozygous CDAN1 variant combination in a patient with a phenotype consistent with CDA‐1. As the phenotypic boundaries of CDA‐1 continue to expand, clinicians should consider CDA‐1 in the differential diagnosis of unexplained macrocytic anemia, even in the absence of severe anemia.

## 1. Introduction

Congenital dyserythropoietic anemia Type 1 (CDA‐1) is a rare hereditary disorder of erythropoiesis. CDA‐1 is inherited in an autosomal recessive manner and is caused by biallelic mutations in either CDAN1 or CDIN1 [1], which encode proteins critical for DNA repair and chromatin reassembly following DNA replication [2–4]. CDA‐1 occurs in all ethnic groups with comparable frequency [5], and diagnosis is based on marrow morphology and genetic analysis identifying a CDAN1 or CDIN1 mutation. Marrow findings include erythroid hyperplasia and dysplasia, increase in binucleate erythroblasts, and the presence of chromatin bridges linking two nuclei [5]. CDA‐1 is linked with high rates of distal skeletal and skin dysmorphic features and with pulmonary hypertension that may persist past infancy [6–8]. The clinical presentation of CDA‐1 is variable; while it commonly presents in the neonatal period or childhood with moderate to severe macrocytic anemia, milder and atypical presentations are increasingly recognized, highlighting the importance of maintaining clinical suspicion across a broad phenotypic spectrum [9]. We describe a patient with findings suggestive of CDA‐1 who presented with mild macrocytic anemia at age 24 and was discovered to have p.(D1043V) and p.(S1036F) mutations, which are currently classified as variants of uncertain significance.

## 2. Case

### 2.1. History

A 24‐year‐old woman was referred to hematology for macrocytic anemia. She was noted to have macrocytic anemia since age 16, with a hemoglobin of 11 g/dL (11.2–16.0 g/dL) and a mean corpuscular volume (MCV) of 102 fL (79–95 fL). Five years later, her hemoglobin was 12.2 g/dL with an MCV of 102 fL. She was then referred to hematology 3 years later, at which time her hemoglobin was 12.1 g/dL and MCV was 103 fL. The remainder of the complete blood count was normal, and at the time of evaluation, she denied blood loss, bruising, delayed hemostasis, or dark urine. She had no family history of anemia, and she experienced menarche at age 14 with intermittently heavy menses that resolved with oral contraceptive use. Surgical history was notable for cholecystectomy at age 13 for biliary colic. Despite cholecystectomy, her total bilirubin remained elevated at age 15 at 2.3 mg/dL (0.1–1.2 mg/dL) with direct bilirubin 0.3 mg/dL (0.0–0.3 mg/dL), which persisted in subsequent years.

### 2.2. Investigations

Additional workup was most notable for evidence of hemolysis and iron overload, with undetectable haptoglobin (30–200 mg/dL), a lactate dehydrogenase of 234 U/L (118–225 U/L), a reticulocyte production index of 2.5 (normal limit in individuals with anemia of 2–3), a ferritin of 749 ng/mL (10–120 ng/mL), and a transferrin saturation of 62% (15%–50%). Vitamin B12, folate, thyroid function, and liver function testing were all within normal range, except for the mildly elevated total bilirubin. Red cell direct antiglobulin, cold agglutinin, paroxysmal nocturnal hemoglobinuria, osmotic fragility, hemoglobin electrophoresis, glucose‐6‐phosphate dehydrogenase, and *HFE* gene testing were normal.

**FIGURE 1:**
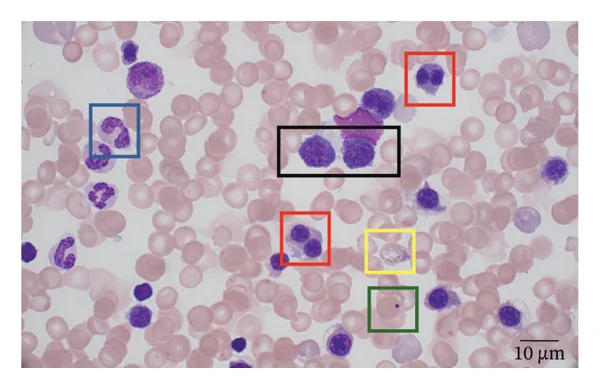
Marrow aspirate smear, hematoxylin and eosin stain, 500x magnification. The smear demonstrates erythroid dysplasia with two binucleate erythroblasts identified in red. A single erythroblast with a dumbbell‐shaped nucleus is labeled in blue, reflecting abnormal nuclear segmentation. Four erythroblasts with cytoplasmic vacuoles are labeled in black, and cytoplasmic vacuolization is noted in surrounding cells. An erythrocyte with a Howell–Jolly body is identified in green, consistent with ineffective erythropoiesis and reduced splenic function in the setting of splenomegaly. A heavily stippled red cell labeled in yellow is also present, reflecting abnormal RNA retention. Several reticulocytes are additionally noted, consistent with a compensatory erythroid response.

**FIGURE 2:**
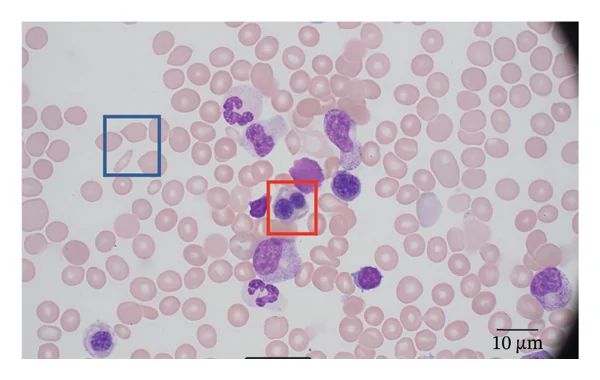
Marrow aspirate smear, hematoxylin and eosin stain, 500x magnification. The smear demonstrates additional dyserythropoietic features consistent with those seen in Figure 1. A binucleate erythroblast is identified in red, supporting the presence of abnormal erythroid maturation. Poikilocytosis is present and labeled in blue, reflecting the heterogeneous red cell morphology characteristic of ineffective erythropoiesis. Marked anisocytosis is noted with the presence of macrocytes and microcytes, consistent with disordered erythroid development. Occasional reticulocytes are also identified, indicating a compensatory erythroid response to chronic anemia.

Physical examination was unremarkable. MRI with T2 iron quantification found 1.6 mg Fe/g dry weight liver (normal < 2.0 mg Fe/g dry weight liver) and revealed hepatomegaly of 20.7 cm and splenomegaly of 17.5 cm in length. Due to persistent anemia with Hgb 10.8 g/dL and MCV 105 fL, a marrow biopsy was performed revealing a normocellular marrow with maturing trilineage hematopoiesis but with dyserythropoietic changes in the form of binucleate erythroblasts, comprising approximately 1% of marrow erythroblasts (Figures 1 and 2). Ovalocytes and spherocytic elliptocytosis were also present in addition to internuclear chromatin bridges.

### 2.3. Genetics

Given the negative studies for common causes of chronic hemolytic anemia, dyserythropoietic changes seen on marrow analysis, and a lack of family history suggesting an inherited marrow failure syndrome, genetic sequencing for hereditary hemolytic anemias was pursued at the Mayo Clinic Laboratories via the Hereditary Hemolytic Anemia Gene Panel, Next Generation Sequencing (Test ID: NHHA). Using this test, DNA extracted from blood undergoes hybrid capture–based library preparation followed by next‐generation sequencing (NGS) with downstream bioinformatics analysis. NGS and/or Sanger sequencing are used to identify variants in coding regions and intron–exon boundaries, with sequence alignment to the GRCh37/hg19 reference genome; all coding exons of CDAN1 (NM 138477.2; Exons 1–28) are analyzed, with supplemental confirmatory sequencing performed as needed.

**FIGURE 3:**
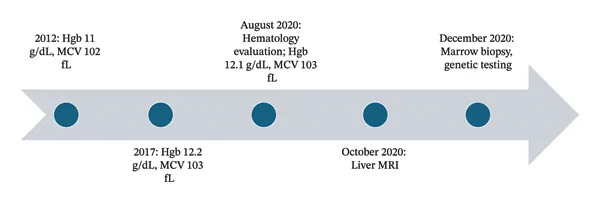
Timeline of workup.

This revealed two mutations in the CDAN1 gene, with Chr15(GRCh37):g.43018584T > A; c.3128A > T, p.(D1043V) and Chr15(GRCh37):g.43018605G > A; c.3107C > T, p.(S1036F) heterozygosity. The genetic sequencing report described these mutations as variants of uncertain significance, noting that no functional studies had been performed to demonstrate that these alterations cause the CDA‐1 phenotype; however, an in silico meta‐predictor suggested that these amino acid changes may impact protein function. Given that this genetic region has been described as mutated in the seminal paper describing CDAN1 mutations as causative of CDA‐1 [1], the patient’s macrocytic anemia and dyserythropoiesis were considered suggestive of CDA‐1. A complete timeline of her workup is available in Figure 3.

## 3. Discussion

CDA‐1 is classically caused by biallelic CDAN1 mutations and may present with transfusion‐dependent anemia during the perinatal period [6–8]. As of 2019, 56 causative mutations have been documented, with a subset of CDA‐1 cases involving mutations in CDIN1 [3]. The clinical presentation of CDA‐1 may differ significantly between patients, who may have variable degrees of macrocytic anemia, hyperbilirubinemia, and hyperferritinemia related to increased destruction of erythroid precursors, although regular red cell transfusions are typically not required [10]. This case is consistent with the notion that CDA‐1 is a genetically heterogeneous condition [9] and corroborates a prior review [11] in which both p.(D1043V) and p.(S1036F) were referenced among causative CDAN1 mutations. Notably, while p.(D1043V) has been previously studied in laboratory‐based functional analyses [12], this report represents the first clinical characterization of this specific compound heterozygous combination in a patient with a phenotype consistent with CDA‐1.

The pathophysiology of CDA‐1 results from the interruption of the binding of CDIN1 with Codanin‐1, leading to impaired chromatin assembly during DNA replication and diminished erythroid maturation [1, 4, 9, 13]. The p.(D1043V) mutation has been shown not to reduce Codanin‐1 protein or mRNA levels, suggesting a functional defect without protein destabilization rather than a loss‐of‐function mechanism [9]. A recent structural and functional analysis of the CDIN1–Codanin‐1 complex discovered three mutations, p.(S1026F), p.(R1042W), and p.(D1043V), that when introduced into Codanin‐1 led to no detectable interaction with CDIN1 [12]. This substantiates prior work [11] suggesting that p.(D1043V) and p.(S1036F) mutations may disrupt interaction between CDIN1 and Codanin‐1. While direct functional studies were not performed in this case, these published findings raise the possibility that the combination of p.(D1043V) and p.(S1036F) mutations may interfere with formation of the CDIN1–Codanin‐1 complex, potentially contributing to a phenotype consistent with CDA‐1.

At present, both p.(D1043V) and p.(S1036F) are currently listed in the National Center for Biotechnology Information ClinVar database as variants of uncertain significance [14]. To further evaluate the pathogenic potential of these variants, both were queried in the Genome Aggregation Database (gnomAD v4; exomes and genomes) [15]. The p.(D1043V) and p.(S1036F) variants were identified with allele frequencies of 0.00004691 and 0.000005019, respectively, with neither observed in a homozygous state across over 1.5 million alleles, supporting their rarity and consistency with a recessive disease mechanism. Population‐specific analysis within gnomAD revealed that p.(D1043V) was most frequently observed in European populations at an allele frequency of 0.0006223. p.(S1036F) was identified in European, Admixed American, and Middle Eastern populations at frequencies of 0.000004272, 0.00003514, and 0.0001653, respectively. Notably, neither variant was observed in a homozygous state in any population, consistent with a recessive disease mechanism.

Conservation analysis using the VarSome variant interpretation platform [16] demonstrated strong evolutionary constraint at both positions. PhyloP scores were 4.5 and 5.009 for D1043 and S1036, respectively, indicating significant conservation across species, as positive values reflect evolutionary constraint. PhastCons scores of 0.919 and 1.000, respectively, further support this, reflecting a 91.9% and 100% probability that these positions fall within conserved genomic elements, suggesting that substitutions at these sites are likely functionally deleterious. In silico prediction using the Franklin Genoox platform [17] further supported the pathogenic potential of p.(D1043V) with an aggregated deleteriousness score of 0.856, while p.(S1036F) returned insufficient data for prediction, as is expected for exceedingly rare variants with limited representation in prediction databases. Taken together, the rarity of both variants, absence of homozygotes, strong evolutionary conservation, and pathogenic prediction for p.(D1043V) are consistent with a recessive disease mechanism supportive of CDA‐1.

Formal variant classification using the American College of Medical Genetics and Genomics and the Association for Molecular Pathology (ACMG/AMP) criteria [18] provides additional context for interpreting these findings. For p.(D1043V), the following criteria are applicable: PM2 (extremely low allele frequency in gnomAD), PP3 (concordant pathogenic in silico prediction), PM1 (location within the critical CDIN1‐binding domain of Codanin‐1), and PP4 (phenotype highly specific for a disorder with a known genetic basis). Based on available evidence, our independent application of ACMG/AMP criteria is suggestive of a likely pathogenic classification for p.(D1043V), though this does not constitute an official ClinVar reclassification. For p.(S1036F), PM2 is applicable given its extreme rarity; however, PP3 cannot be applied due to insufficient in silico prediction data; therefore, p.(S1036F) remains a variant of uncertain significance. A significant limitation of this case is that parental testing was not performed to confirm that the variants are in trans; therefore, the possibility that both exist in cis cannot be excluded. Confirmation that the variants are located on opposite alleles would substantially strengthen the evidence for a compound heterozygous autosomal recessive disease mechanism, while identification of both variants in cis would necessitate reassessment of the diagnosis. Parental genetic testing should therefore be strongly considered in future clinical encounters involving these or similar variants, and this case should be interpreted with this limitation in mind.

The diagnosis of CDA‐1 may be reinforced by marrow analysis via both light and electron microscopy. Typically, light microscopy reveals dyserythropoietic features including erythroid hyperplasia, binuclear erythroblasts, and macrocytosis as in this case (Figures 1 and 2), although these findings are nonspecific. In this case, binucleate erythroblasts comprised approximately 1% of erythroblasts, below the commonly cited diagnostic threshold of 2%–3%, which is consistent with the mild phenotypic presentation observed. More diagnostically relevant was the presence of internuclear chromatin bridges on light microscopy, which may be present in up to 2.8% of erythroblasts [1] and are more specific for the diagnosis [19]. Additionally, electron microscopy may reveal a characteristic “spongy” or “Swiss cheese” appearance of heterochromatin in erythroid precursors; however, electron microscopy was not performed in favor of molecular testing. Notably, a 2024 study of 36 patients with clinical suspicion of CDA‐1 demonstrated that morphological features traditionally considered characteristic of CDA‐1, including binucleated normoblasts and internuclear bridges, can also be present in other hereditary anemias such as pyruvate kinase deficiency, underscoring the essential role of genetic testing in establishing a definitive diagnosis [20].

In the evaluation of macrocytic anemia, the differential diagnosis should include megaloblastic anemia, autoimmune hemolytic anemia, paroxysmal nocturnal hemoglobinuria, hereditary spherocytosis, glucose‐6‐phosphate dehydrogenase deficiency, hypothyroidism, liver dysfunction, alcohol use, hereditary hemochromatosis, and medications such as hydroxyurea or methotrexate. In this case, each was systematically excluded through laboratory testing and clinical history, directing the workup toward genetic testing for a congenital dyserythropoietic etiology. Multiple reports in the literature have demonstrated that anemia in CDA‐1 is not always severe, and mild or asymptomatic presentations should not preclude consideration of this diagnosis [20, 21]. Notably, the degree to which CDA‐1–associated mutations disrupt the CDIN1–Codanin‐1 interaction has been shown to vary by mutation, potentially contributing to the phenotypic variability observed across patients [22]. When genetic testing is unavailable or inconclusive, alternative diagnoses including myelodysplastic syndrome, pyruvate kinase deficiency, sideroblastic anemia, and other congenital anemias should remain on the differential.

The management of CDA‐1 is based on the severity of anemia. Mild phenotypes are typically managed with surveillance and transfusion when necessary, whereas severe phenotypes may require interferon alpha therapy to reduce transfusion dependence [23]. In addition to close monitoring of hemoglobin, iron, transferrin, ferritin, and bilirubin, individuals with CDA‐1 are at risk for iron overload and are advised to undergo annual myocardial and liver T2‐weighted MRI, abdominal ultrasound, and visual acuity testing, as well as regular bone densitometry testing [1]. Splenectomy has not been shown to consistently improve anemia in individuals with CDA‐1 [10], and in this case, iron chelation was deferred given the absence of hepatic iron overload on imaging or other organ dysfunction indicative of iron overload and a ferritin value of less than 1000 ng/mL.

## 4. Conclusion

This case describes the compound heterozygous mutations p.(D1043V) and p.(S1036F), which are currently classified as variants of uncertain significance, identified in a patient with mild macrocytic anemia suggestive of CDA‐1. When considered alongside a recent functional and structural analysis of the CDIN1–Codanin‐1 complex, this report provides clinical evidence supporting the possibility that these variants may contribute to the observed CDA‐1 phenotype. Given the possibility of both variants occurring in cis in this case, further work including segregation studies and functional assays will be required to definitively establish the pathogenic significance of this variant combination. Ultimately, this case contributes to the growing recognition of the phenotypic and genetic heterogeneity of CDA‐1.

## Author Contributions

All authors made individual contributions to this manuscript. Kevin G. Zablonski wrote the original manuscript draft and manuscript revisions. Neil A. Lachant oversaw the diagnosis and management of the patient; however, he passed away before the final draft of the manuscript was completed. Archibald Perkins provided bone marrow analysis and images. William J. Archibald assisted with the original manuscript draft and manuscript revisions.

## Funding

The authors received no specific funding for this work.

## Disclosure

All authors have read and approved the final version of the manuscript. Kevin G. Zablonski had full access to all of the data in this study and takes complete responsibility for the integrity of the data and the accuracy of the data analysis.

## Consent

The patient granted informed consent for the publication of this case.

## Conflicts of Interest

The authors declare no conflicts of interest.

## Supporting Information

Additional supporting information can be found online in the Supporting Information section.

## Supporting information


**Supporting Information** CARE checklist—Case Reports in Hematology.

## Data Availability

Data sharing is not applicable to this article as no datasets were generated or analyzed during the current study.
