# Supplementary material for: Double Heterozygous CDAN1 Variants of Uncertain Significance Associated With a Phenotype Consistent With Congenital Dyserythropoietic Anemia Type 1
Source: Case Rep Hematol. 2026 Aug 31;2026:8073716. doi: 10.1155/crh/8073716 (PMC13530274; doi:10.1155/crh/8073716)
Supplement: Supplementary file 1 [file CRH-2026-8073716-s001.docx]

CARE Checklist of information to include when writing a case report

**Topic	Item	Checklist item description	Reported on Line**

**Title	1**	The diagnosis or intervention of primary focus followed by the words “case report” . . . . . . . . . . . . . . . . . .	1-2

**Key Words	2**	2 to 5 key words that identify diagnoses or interventions in this case report, including "case report"	18

**Abstract**

**(no references)**

**3a**	Introduction: What is unique about this case and what does it add to the scientific literature?	27-28

**3b**	Main symptoms and/or important clinical findings . . . . . . . . . . . . . . . . . . . . . . . . . . . . . . . . . . . . . . . . . . . . . . . . . . .	25-26

**3c**	The main diagnoses, therapeutic interventions, and outcomes	24-28

**3d**	Conclusion—What is the main “take-away” lesson(s) from this case?	27-28

**Introduction	4**	One or two paragraphs summarizing why this case is unique (**may include references**)	53-66

**Patient Information	5a**	De-identified patient specific information	69-70

**5b**	Primary concerns and symptoms of the patient	69

**5c**	Medical, family, and psycho-social history including relevant genetic information	73-77

**5d**	Relevant past interventions with outcomes	69-87

**Clinical Findings**

**Timeline**

**Diagnostic Assessment**

**Therapeutic Intervention**

**Follow-up and Outcomes**

6 Describe significant physical examination (PE) and important clinical findings	83-84
7 Historical and current information from this episode of care organized as a timeline	69-99

**8a**	Diagnostic testing (such as PE, laboratory testing, imaging, surveys).	79-99

**8b**	Diagnostic challenges (such as access to testing, financial, or cultural)	N/A

**8c**	Diagnosis (including other diagnoses considered)	82-83

**8d**	Prognosis (such as staging in oncology) where applicable	N/A

**9a**	Types of therapeutic intervention (such as pharmacologic, surgical, preventive, self-care) . . . . . . . . . . . . . . . . .	125-132

**9b**	Administration of therapeutic intervention (such as dosage, strength, duration)	127-132

**9c**	Changes in therapeutic intervention (with rationale)	N/A

**10a**	Clinician and patient-assessed outcomes (if available)	N/A

**10b**	Important follow-up diagnostic and other test results	130-132

**10c**	Intervention adherence and tolerability (How was this assessed?)	N/A

**10d**	Adverse and unanticipated events	N/A

**Discussion	11a**	A scientific discussion of the strengths AND limitations associated with this case report	104-132

**11b**	Discussion of the relevant medical literature **with references**	104-132

**11c**	The scientific rationale for any conclusions (including assessment of possible causes)	135-139

**11d**	The primary “take-away” lessons of this case report (without references) in a one paragraph conclusion	135-139

**Patient Perspective	12**	The patient should share their perspective in one to two paragraphs on the treatment(s) they received . . . .	N/A

**Informed Consent	13**	Did the patient give informed consent? Please provide if requested . . . . . . . . . . . . . . . . . . . . . . . . . . . . . . . . . . . . . .	**Yes** **No**
